# EUS-based intratumoral and peritumoral machine learning radiomics analysis for distinguishing pancreatic neuroendocrine tumors from pancreatic cancer

**DOI:** 10.3389/fonc.2025.1442209

**Published:** 2025-03-04

**Authors:** Shuangyang Mo, Nan Yi, Fengyan Qin, Huaying Zhao, Yingwei Wang, Haiyan Qin, Haixiao Wei, Haixing Jiang, Shanyu Qin

**Affiliations:** ^1^ Gastroenterology Department, The First Affiliated Hospital of Guangxi Medical University, Nanning, China; ^2^ Gastroenterology Department/Clinical Nutrition Department, Liuzhou People’s Hospital Affiliated to Guangxi Medical University, Liuzhou, China

**Keywords:** pancreatic neuroendocrine tumors, peritumoral, endoscopic ultrasonography, radiomics, machine learning, pancreatic cancer

## Abstract

**Objectives:**

This study aimed to develop and validate intratumoral, peritumoral, and combined radiomic models based on endoscopic ultrasonography (EUS) for retrospectively differentiating pancreatic neuroendocrine tumors (PNETs) from pancreatic cancer.

**Methods:**

A total of 257 patients, including 151 with pancreatic cancer and 106 with PNETs, were retroactively enrolled after confirmation through pathological examination. These patients were randomized to either the training or test cohort in a ratio of 7:3. Radiomic features were extracted from the intratumoral and peritumoral regions from conventional EUS images. Following this, the radiomic features underwent dimensionality reduction through the utilization of the least absolute shrinkage and selection operator (LASSO) algorithm. Six machine learning algorithms were utilized to train prediction models employing features with nonzero coefficients. The optimum intratumoral radiomic model was identified and subsequently employed for further analysis. Furthermore, a combined radiomic model integrating both intratumoral and peritumoral radiomic features was established and assessed based on the same machine learning algorithm. Finally, a nomogram was constructed, integrating clinical signature and combined radiomics model.

**Results:**

107 radiomic features were extracted from EUS and only those with nonzero coefficients were kept. Among the six radiomic models, the support vector machine (SVM) model had the highest performance with AUCs of 0.853 in the training cohort and 0.755 in the test cohort. A peritumoral radiomic model was developed and assessed, achieving an AUC of 0.841 in the training and 0.785 in the test cohorts. The amalgamated model, incorporating intratumoral and peritumoral radiomic features, exhibited superior predictive accuracy in both the training (AUC=0.861) and test (AUC=0.822) cohorts. These findings were validated using the Delong test. The calibration and decision curve analyses (DCA) of the combined radiomic model displayed exceptional accuracy and provided the greatest net benefit for clinical decision-making when compared to other models. Finally, the nomogram also achieved an excellent performance.

**Conclusions:**

An efficient and accurate EUS-based radiomic model incorporating intratumoral and peritumoral radiomic features was proposed and validated to accurately distinguish PNETs from pancreatic cancer. This research has the potential to offer novel perspectives on enhancing the clinical utility of EUS in the prediction of PNETs.

## Introduction

Pancreatic neuroendocrine tumors (PNETs) are uncommon tumors derived from endocrine cells in pancreatic islet tissues and constitute approximately 3% of all pancreatic neoplasms (1). PNETs are the second most prevalent pancreatic neoplasms and are broadly categorized into functional (hormone-producing) and nonfunctional tumors (2, 3). Compared with functional PNETs, nonfunctional PNETs have a greater incidence and a more disadvantageous prognosis (4). The preoperative identification of PNETs is a critical challenge in clinical practice, as PNETs rely primarily on pathological examination and immunohistochemistry, with pancreatic cancer being the most essential differential diagnosis (5). The surgical procedure for PNETs differs immensely from the operation for more aggressive pancreatic cancer (6). A pNET with a diameter ≥2 cm has a critical risk of lymph node metastasis (>20%) and should undergo surgical resection (7). Conversely, the strategy of observation represents a more suitable and safer approach for most low-grade PNETs with a diameter <2 cm, given the low risk of metastasis, progression, or morbidity (6, 8). Therefore, accurate preoperative distinction between PNETs and pancreatic cancer is imperative not only for deciding on a more appropriate therapeutic strategy but also for providing patients with a better prognosis (9).

Endoscopic ultrasonography (EUS) is commonly applied to diagnose PNETs. It is regarded as one of the most accurate imaging modalities for diagnosing pancreatic diseases due to its ability to produce high-resolution images of the pancreas and its sensitivity ranging from 57% to 94% (10). As outlined in the 2023 consensus guidelines of the European Neuroendocrine Tumor Society (ENETS), EUS is deemed the preferred imaging modality in cases where other noninvasive imaging methods have yielded negative results. This preference is due to EUS’s ability to offer meticulous observation and assessment of PNETs, as well as its capacity to conduct a comprehensive examination of the pancreas (11). Additionally, previous studies have shown that EUS-guided fine-needle aspiration (EUS-FNA) can reliably distinguish the tumor grade of PNETs (12). E EUS holds a significant role in the diagnostic assessment of PNETs because of its high accuracy and sensitivity in clinical and pathological diagnosis. However, PNETs with characteristics such as a measurement greater than 2 cm, anomalous margins, miscellaneous echotexture, and dilatation of the upstream main pancreatic duct are strongly correlated with aggressiveness. They are indistinguishable from most common pancreatic cancer cases (13). Furthermore, the recent EUS-based identification of pancreatic lesions using EUS is primarily based on macroscopic anatomical imaging features, which results in unsatisfactory specificity and is easily influenced by subjective endoscopists.

Currently, integrating radiomic and machine learning strategies based on imaging modalities is recommended for use in cancer differential diagnosis and prognosis prediction. Radiomics facilitates the extraction and analysis of multiple quantitative image features using high-throughput techniques. These features are subsequently utilized to develop diverse tumor diagnosis and prediction models using various machine learning, deep learning, and other algorithmic approaches (14, 15). Numerous studies have evaluated the use of computed tomography (CT), magnetic resonance imaging (MRI), and ultrasonography (US) radiomics in the diagnosis and prognostication of PNETs, revealing the remarkable efficacy of these methods (16–18).

However, most related studies have focused only on the predictive radiomic features of intratumoral lesions, ignoring potential information from peritumoral lesions and surrounding tissues. Previous reports have demonstrated the prominent diagnostic performance of peritumoral regions in imaging-based radiomics and indicated that peritumoral regions contain vast quantities of information correlated with tumor characteristics (19, 20). Bence S reported that carefully identifying the characteristics of peritumoral pancreatic regions, specifically the morphology and hormone expression profile of endocrine islets, may improve the accuracy of classifying hereditary and sporadic PNETs and contribute to patient treatment strategies and prognosis (21). Similarly, Xie N suggested that combined peritumoral and intratumoral texture multiparametric MRI-based radiomic features achieved greater accuracy in preoperatively assessing the pathological outcomes of pancreatic cancer (22).

Despite the recognized effectiveness of EUS as an imaging modality, the utility of EUS-based radiomics in improving the identification of PNETs from pancreatic cancer has not been confirmed. Leveraging existing knowledge, we utilized various common machine learning algorithms to develop and validate a robust radiomic model based on intratumoral and peritumoral radiomic features for the differentiation of PNETs from pancreatic cancer.

## Materials and methods

### Study population

The institutional ethics review board of the First Affiliated Hospital of Guangxi Medical University approved this retrospective study (No. 2023-K346-01), thereby exempting the need for patient consent or signed informed consent for the examination of medical images and clinical information. A cohort of 257 patients with pancreatic tumors, comprising 151 individuals with pancreatic cancer and 106 with PNETs, underwent pancreatic surgery or endoscopic ultrasonography-guided fine-needle aspiration/biopsy (EUS-FNA/B) at our institution from October 2012 to October 2023, were enrolled in this research. The criteria for inclusion and exclusion are delineated herein.

**Figure 1:**
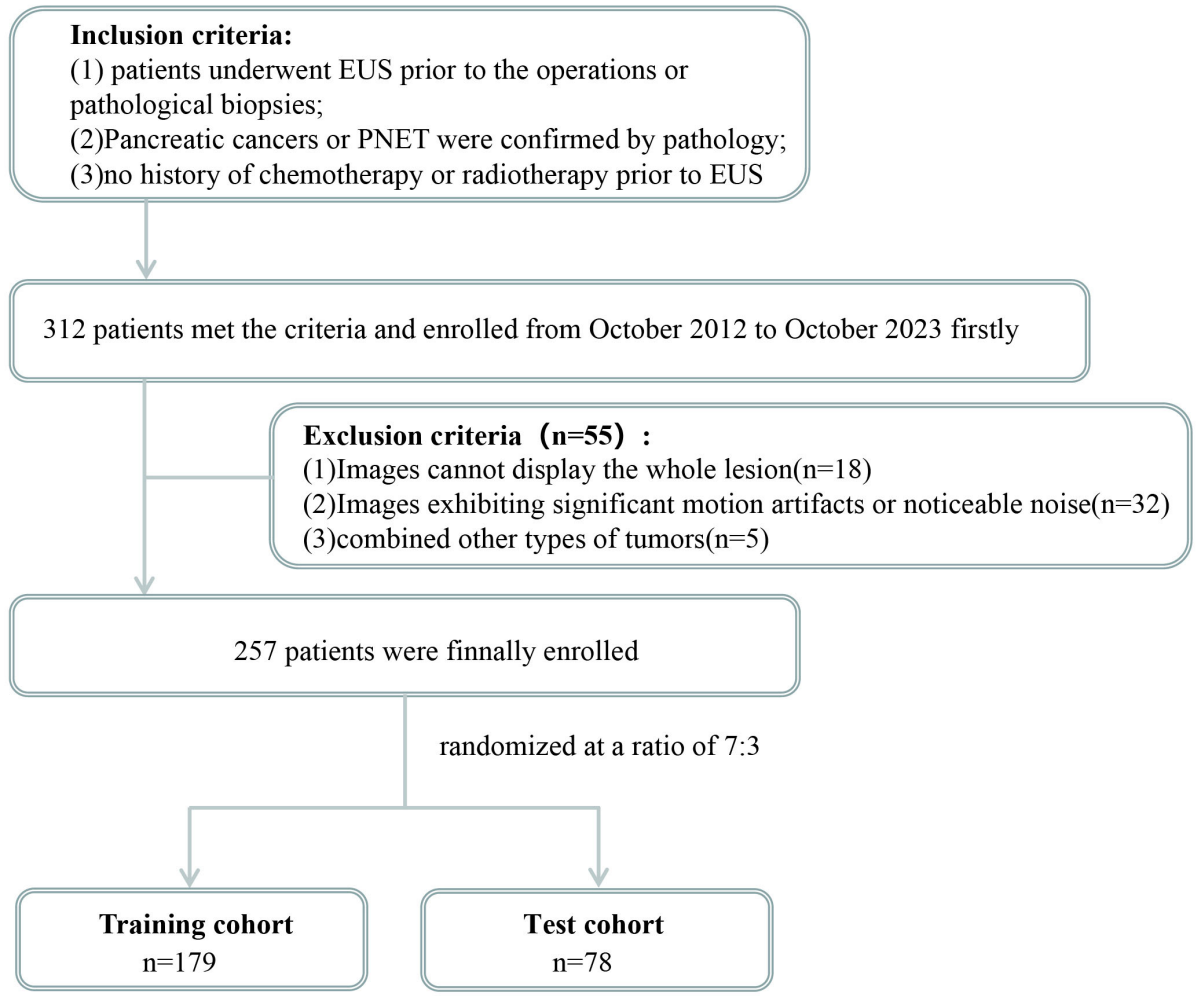
Flowchart for enrolling the study population.

The inclusion criteria for patients were as follows (1): underwent preoperative EUS scan of the pancreas meticulously; (2) had pancreatic cancer or PNET confirmed by postoperative pathology or EUS-FNA pathology; and (3) had complete and clear EUS images available before the patient’s preoperative or pathological biopsies. (4) Patients who could not receive any chemotherapy or radiotherapy before EUS. The exclusion criteria for patients were as follows: (1) inability to display the whole lesion; (2) significant motion artifacts or noticeable noise; and (3) the presence of other types of tumors. The patients were allocated randomly into a training cohort and a test cohort at a ratio of 7:3, as depicted in **Figure 1**.

In this study, a retrospective analysis was performed on a range of clinical parameters, including age, sex, location of the pancreatic mass, CA-199 levels, pathological diagnosis, and EUS characteristics of pancreatic lesions. Additionally, univariate and multivariate logistic regression analyses were conducted on each clinical parameter to identify statistically significant clinical features. Forest plots for univariate and multivariate logistic regression analyses were plotted using online tools (http://www.bioinformatics.com.cn).

### EUS image acquisition

All enrolled patients underwent preoperative or pre-biopsy endoscopic ultrasound (EUS) examinations of the pancreas using FUJIFILM SU-9000 and Olympus EU-ME2 equipment at the First Affiliated Hospital of Guangxi Medical University. As the largest EUS medical center in the Guangxi Zhuang Autonomous Region, the facility performs over 3,000 EUS diagnostic and therapeutic procedures annually. To ensure the consistency of operating procedures, a highly experienced EUS specialist, with over 30,000 EUS procedures, conducted a comprehensive examination of the pancreatic region and acquired detailed images of the masses with the same EUS procedures. These images consistently employed a grayscale level of 125 values and a grayscale window of 250 values. The imaging data was obtained by accessing information from our institutional Picture Archive and Communication System (PACS).

### ROI delineation

**Figure 2:**
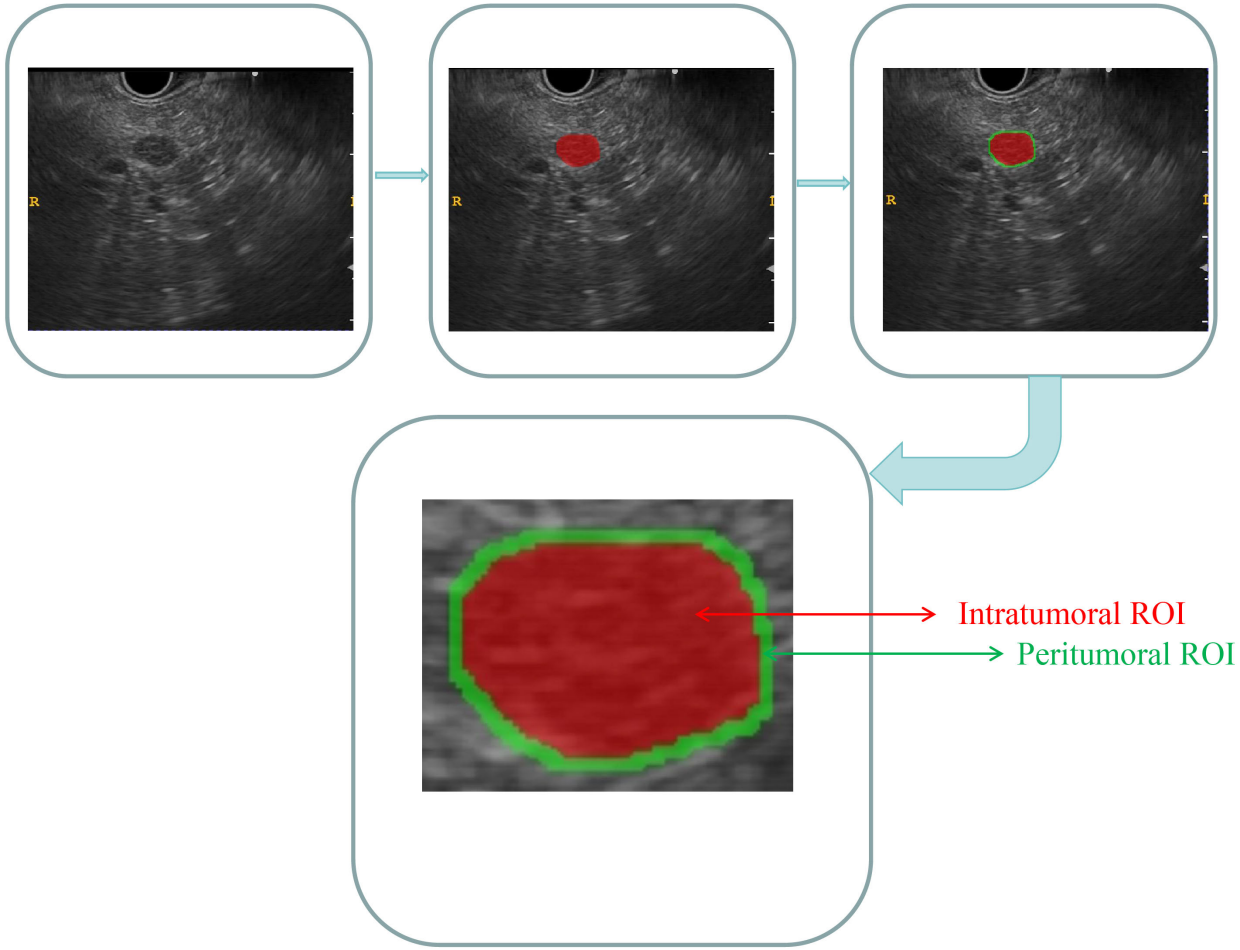
Comprehensive diagram of the intratumoral and peritumoral ROIs. Red indicates the “intratumoral ROI”; green indicates the “peritumoral ROI”.

As the case had already been reviewed by the EUS specialist who initially acquired the images, the DICOM-formatted images were subsequently analyzed by two experienced EUS specialists, each with 6 and 7 years of expertise. These specialists manually delineated the intratumoral region of interest (ROI) using ITK-SNAP software. (version 3.8.1, http://www.itksnap.org). Discrepancies in the specialists’ delineations were resolved through collaborative discussion and consensus. Both specialists were blinded to the patients’ histopathological diagnoses. The lesions were meticulously delineated carefully along the margins on conventional EUS images. The peritumoral ROI was acquired by expanding the intratumoral region of interest (ROI) delineation by 3 mm via the use of a regular morphological dilation procedure with ITK-SNAP software. Finally, for each EUS image, three dissimilar ROI images were acquired: an intratumoral ROI, a peritumoral ROI, and a combined ROI that integrated both the intratumoral and peritumoral ROIs. A comprehensive diagram can be found in **Figure 2**.

Standardization techniques were utilized for preprocessing the images and data to ensure the reproducibility of the findings. The intraclass correlation coefficient (ICC) was used to evaluate both intraobserver and interobserver reproducibility. A random selection of one-third of the participants resulted in 85 patients, including 63 with pancreatic cancer and 22 with PNETs, was randomly selected. Following a two-week interval, the same EUS specialists conducted intratumoral ROI segmentation again. An ICC value greater than 0.9 signified a high level of agreement.

### Radiomics feature extraction

The categorization of handcrafted features can be delineated into three discrete groups, namely intensity, geometric, and textural. Geometric features are concerned with the three-dimensional morphological characteristics of tumors. Intensity features refer to the statistical dispersion of voxel intensities within the tumor at the first order. Contrarily, textural features elucidate patterns and higher-order spatial distributions of intensities. This article employed a variety of methodologies, such as the gray level co-occurrence matrix (GLCM), gray level run length matrix (GLRLM), and gray level size zone matrix (GLSZM), and neighborhood gray-level difference matrix (NGTDM), to extract texture features. A list of all the radiomics features covered in this project is provided in **Supplementary Data 1**.

The procedures for extracting the radiomic features of the intratumoral and peritumoral ROIs were performed separately. Furthermore, the radiomic features of the combined ROIs were obtained through the integration of features from both intratumoral and peritumoral ROIs.

The algorithms employed to extract radiomic features were based on the Image Biomarker Standardization Initiative (IBSI) (23).

### Radiomic feature selection

To evaluate the reliability of these radiomic features, a Mann−Whitney *U* test was performed to compare the PNETs and pancreatic cancer cohorts, followed by feature selection. Only radiomic features with a significance level of *P*<0.05 were retained for further analysis. Spearman’s rank correlation coefficient was then utilized to assess the interrelationship between each feature, ensuring the robustness of the analysis (**Supplementary Figures 1**-**3**). Any feature with a correlation coefficient greater than 0.9 between any two features was retained with one of them, randomly. Additionally, a greedy recursive deletion strategy was employed to enhance feature representation by iteratively removing the most redundant feature within the current set.

Subsequently, a 10-fold cross-validation technique was utilized to identify features with nonzero coefficients through the application of the LASSO regression model. Notably, the penalty parameter (lambda.min) was determined by the minimum criterion. The feature-selecting procedures were carried out within the training cohort and eventually implemented in the test cohort. Features with nonzero coefficients were preserved for the regression model fitting process and amalgamated into a radiomic signature.

The radiomic scores for each patient were determined by applying a linear combination of the retained features and their corresponding model coefficients. The LASSO regression analysis was performed utilizing the Python scikit-learn package.

### Construction of radiomic models

Multiple prevailing machine learning algorithms were applied to construct categorization models for the optimal identification of pancreatic cancer and PNETs. After LASSO feature filtering was applied, the selected intratumoral ROI radiomic features were input into commonly utilized machine learning models, including logistic regression (LR), random forest (RF), XGBoost, support vector machine (SVM), extra tree, and MLP models, for the development of intratumoral radiomic models. To achieve optimal model performance and mitigate the risk of overfitting, we conducted hyperparameter tuning. The pertinent parameters and hyperparameter space for each model are detailed in **Supplementary Data 2**.

The ultimate radiomic signature was determined through the application of a 5-fold cross-validation method. The diagnostic performance of various machine learning algorithms was evaluated through the use of metrics such as the area under the receiver operating characteristic curve (AUC), accuracy, specificity, sensitivity, positive predictive value (PPV), and negative predictive value (NPV), leading to the identification of the most optimal intratumoral radiomic model. In this study, we employed 2000 bootstrap samples to determine the 95% confidence intervals (CI) for the AUC.

Subsequently, the selected machine learning algorithm, which achieved reasonable performance, was applied to establish peritumoral and combined radiomic models.

### Radiomics model assessment and radiomics signature definition

Based on the consistent machine learning algorithm, an intratumoral radiomic model, peritumoral radiomic model, and combined radiomic model were constructed. The ROC curves were conducted to evaluate the diagnostic effectiveness of these three radiomic models in both the training and test cohorts. Following this, a Delong test was performed to compare their performance, in terms of the AUC.

The concordance between the predictions of these diverse radiomic models and the observed outcomes was evaluated through the calculation of calibration curves, which juxtaposed the predictions of the models with the actual observations. The calibration efficiency of these three radiomic models was assessed through the construction of calibration curves, while the Hosmer–Lemeshow (H-L) analytical fit was applied to assess the calibration capability of these radiomic models. Furthermore, decision curve analysis (DCA) was employed to assess the clinical efficacy of the predictive models. Finally, the best performing radiomics model was defined as the radiomics signature.

### Construction of clinical signature and nomogram

The same radiomics signature was utilized to develop the clinical signature using an identical machine learning algorithm. Subsequently, a nomogram was constructed to intuitively and efficiently assess the incremental predictive value of the integrated radiomics and clinical signatures. The performance of the nomogram was then evaluated using a calibration curve, ROC curve, and DCA.

### Statistical analysis

**Figure 3:**
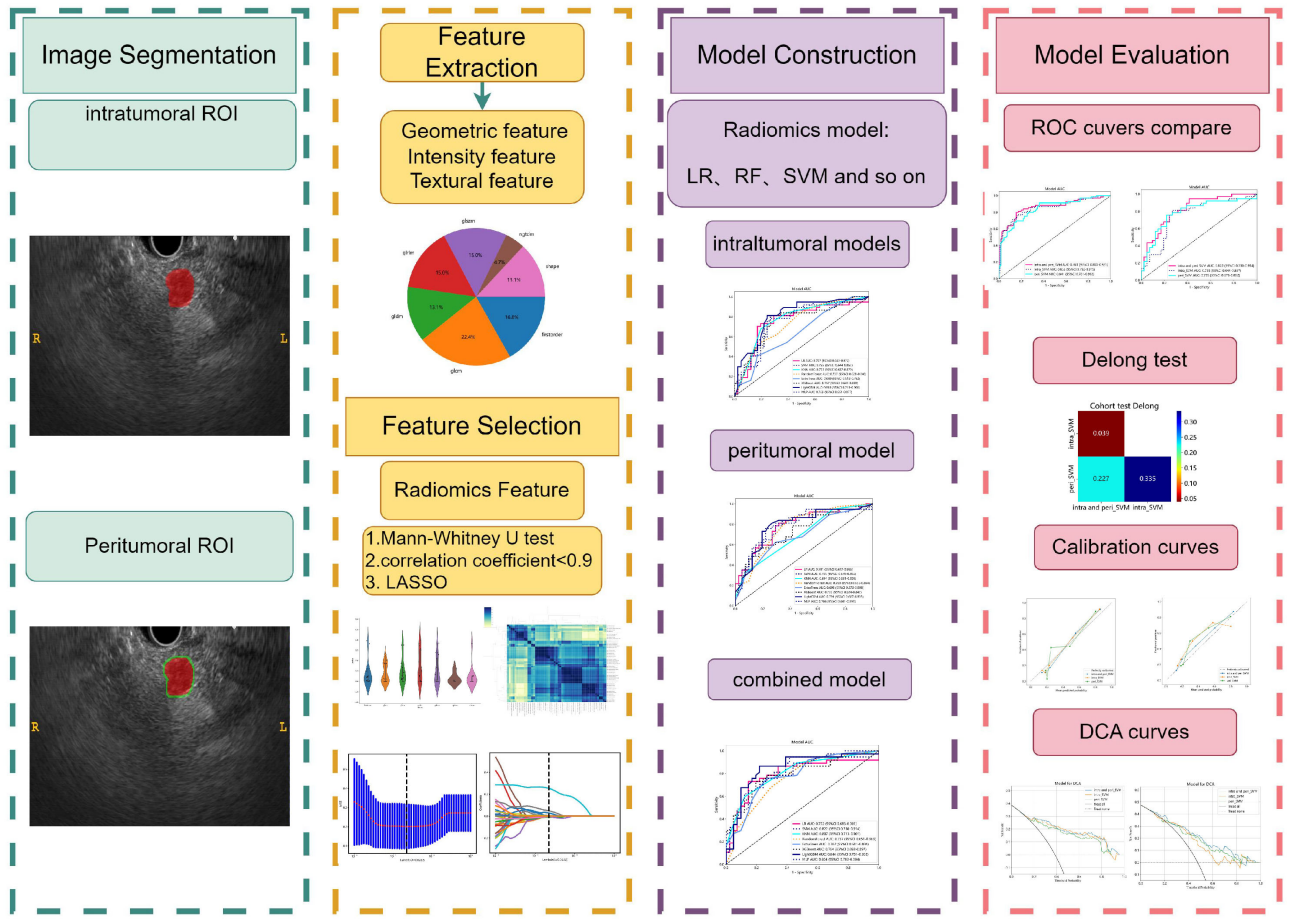
The workflow of this study.

A comparison of clinical parameters and radiomic features among participants was conducted using appropriate statistical tests such as independent sample *t*-test, Mann−Whitney *U* test, or *X^2^* test. Statistical significance was determined at a two-tailed *p*-value < 0.05. Prediction performance was evaluated based on metrics including AUC, accuracy, sensitivity, specificity, PPV, and NPV. The Delong test was conducted to statistically compare the AUC performance between the two models. The comprehensive methodology for this research is illustrated in **Figure 3**.

## Results

### Baseline population characteristics

A total of 257 patients (141 women, 116 men) were enrolled in this retrospective research, containing 179 patients in the training cohort and 78 patients in the test cohort. The results indicated no significant variance in patient age, sex, pathological classification, and location of the pancreatic mass between the training and test cohorts, as detailed in **Supplementary Table 1**. Furthermore, the comparison of clinical parameters between pancreatic cancer and PNETs is detailed in **Table 1**. The results demonstrated significant differences in age, maximum diameter, CA-199 levels, shape, margin characteristics, echo uniformity, and the presence of cystic degeneration between patients with pancreatic cancer and those with PNETs within the training cohort. Conversely, no significant differences were identified concerning gender, pancreatic mass location, and the presence of cystic degeneration in the test cohort.

**Table 1:** Clinical and radiological characteristics in the training and test cohorts.

Variable	Training cohort (N=179)			Test cohort (N=78)		
	Pancreatic cancers	PNETs	P-value	Pancreatic cancers	PNETs	P-value
Age	58.76 ± 9.48	48.13 ± 13.54	<0.001	59.27 ± 10.87	45.30 ± 12.44	<0.001
Maximum diameter	36.45 ± 12.73	21.78 ± 13.75	<0.001	36.23 ± 13.83	21.26 ± 12.89	<0.001
Log (CA-199)			<0.001			<0.001
Gender			0.041			0.543
0	60 (54.55%)	49 (71.01%)		15 (36.59%)	17 (45.95%)	
1	50 (45.45%)	20 (28.99%)		26 (63.41%)	20 (54.05%)	
Shape			<0.001			<0.001
0	85 (77.27%)	22 (31.88%)		32 (78.05%)	12 (32.43%)	
1	25 (22.73%)	47 (68.12%)		9 (21.95%)	25 (67.57%)	
Margin			<0.001			0.013
0	49 (44.55%)	5 (7.25%)		17 (41.46%)	5 (13.51%)	
1	61 (55.45%)	64 (92.75%)		24 (58.54%)	32 (86.49%)	
Echo			0.271			0.011
0	4 (3.64%)	6 (8.70%)		1 (2.44%)	9 (24.32%)	
1	106 (96.36%)	63 (91.30%)		40 (97.56%)	28 (75.68%)	
Uniformity			<0.001			0.014
0	87 (79.09%)	30 (43.48%)		29 (70.73%)	15 (40.54%)	
1	23 (20.91%)	39 (56.52%)		12 (29.27%)	22 (59.46%)	
Cystic areas			<0.001			0.086
0	78 (70.91%)	64 (92.75%)		34 (82.93%)	36 (97.30%)	
1	32 (29.09%)	5 (7.25%)		7 (17.07%)	1 (2.70%)	
Location			0.546			0.812
0	51 (46.36%)	28 (40.58%)		21 (51.22%)	17 (45.95%)	
1	59 (53.64%)	41 (59.42%)		20 (48.78%)	20 (54.05%)	

Gender: “0” means female, “1” means male; Shape: “0” means irregular shape, “1” means regular shape; Margin: “0” means unclear margin of lesion, “1” means clear margin of lesion; Echo: “0” means means not hypoechoic of lesion, “1” means hypoechoic of lesion; uniformity: “0” means nonuniformity of echo; “1” means uniformity of echo; Cystic areas: “0” means no cystic areas, “1” means cystic areas; Location: “0” means head and uncinate process of the pancreas, “1” means body and tail of the pancreas.

### Radiomics feature extraction and screening

PyRadiomics, an internal feature analysis tool, extracted all handcrafted features. A thorough analysis was conducted to identify a total of 107 manually derived radiomic features across 7 categories, including 18 first-order features, 14 shape features, and the remaining texture features. Finally, the quantity and classifications of intratumoral and peritumoral radiomic features derived from EUS images demonstrated complete consistency. The combined radiomic features were generated by overlaying the intratumoral and peritumoral radiomic features. Detailed definitions regarding these handcrafted features can be found in these articles (24).

**Figure 4:**
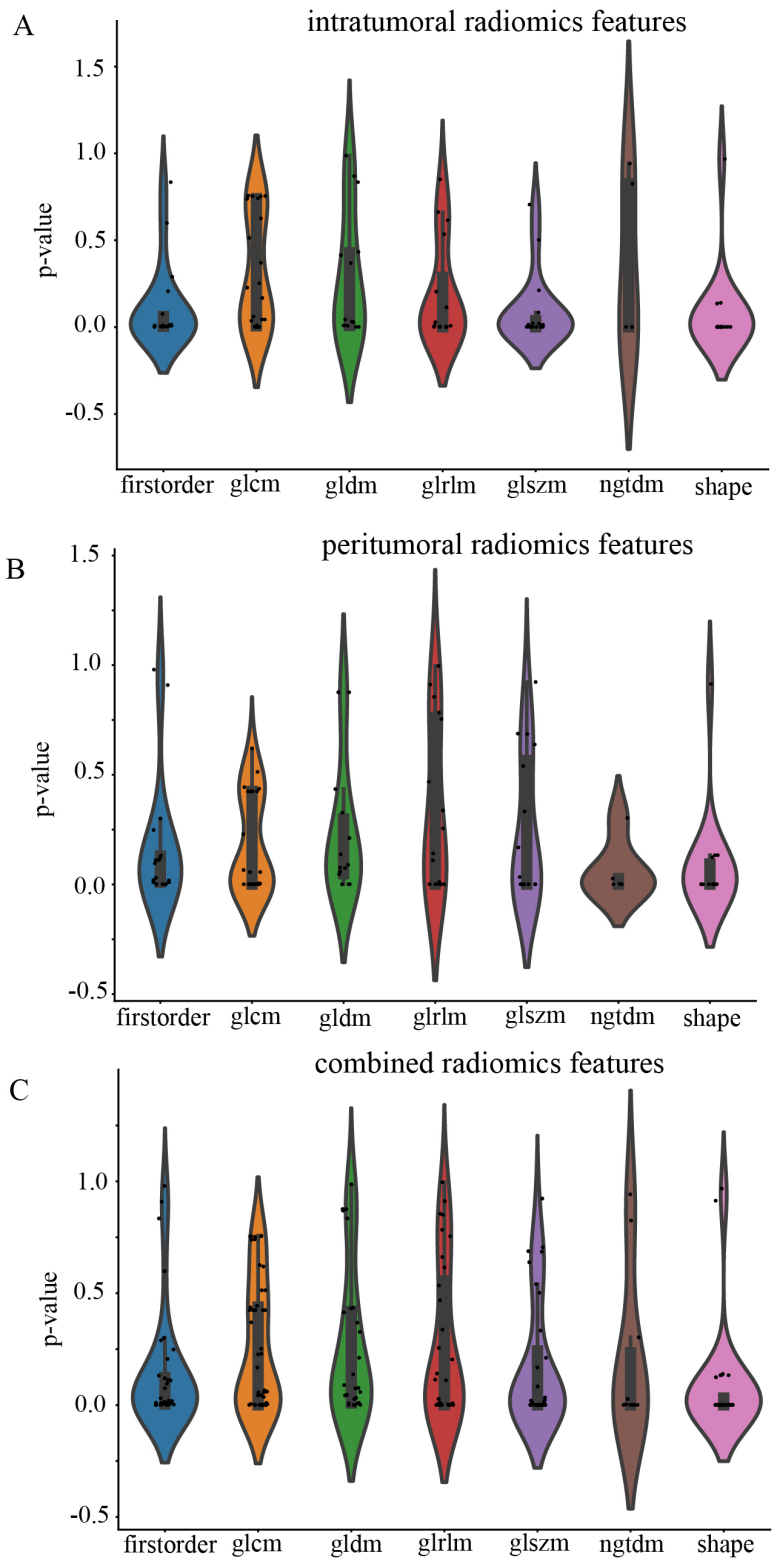
Violin plot for differential analyses of intratumoral **(A)**, peritumoral **(B)**, and combined **(C)** radiomic features with their corresponding p values.

**Figure 5:**
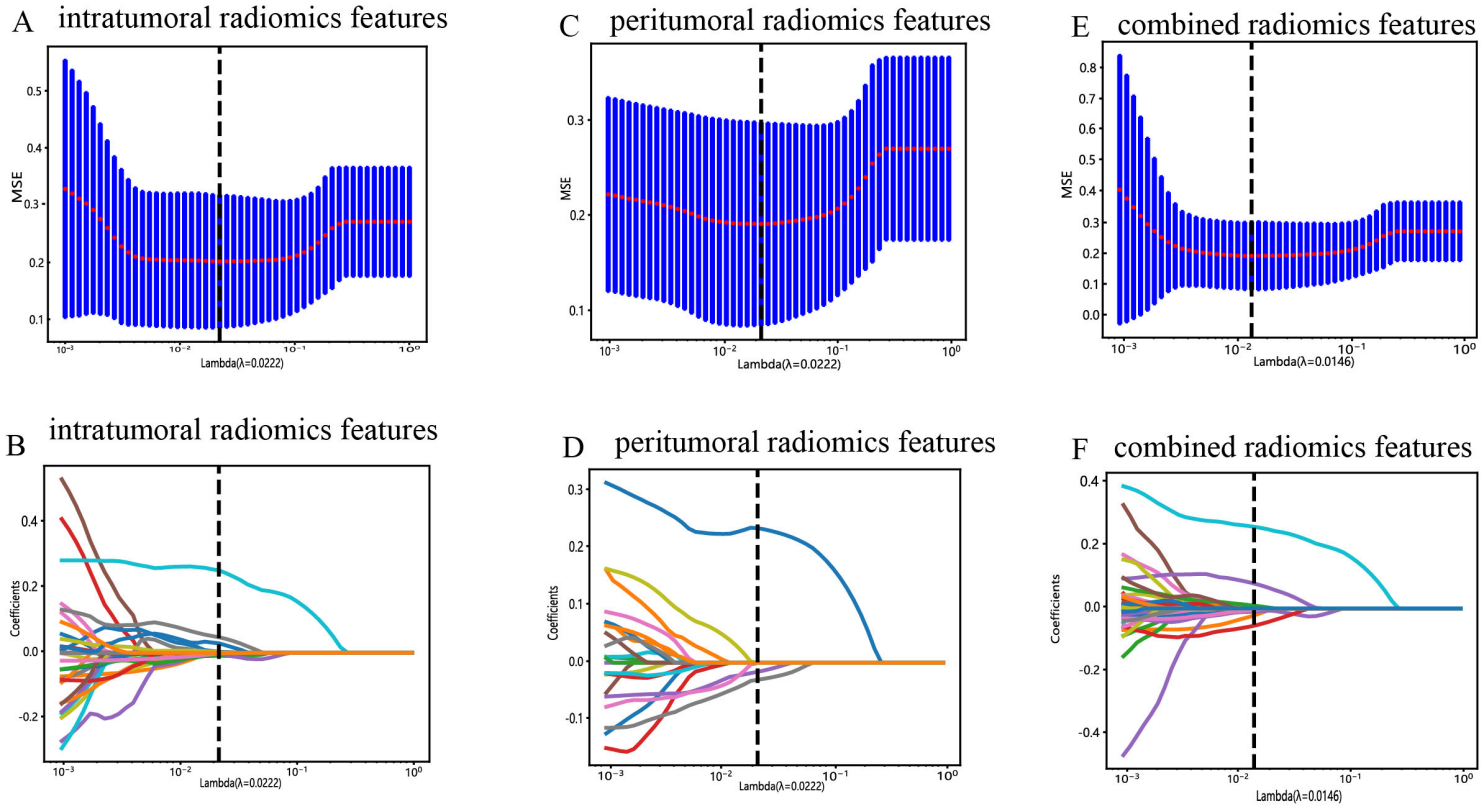
Radiomic feature selection with the LASSO regression model. **(A)** The LASSO model’s tuning parameter (λ) was selected using 10-fold cross-validation via the minimum criterion. The vertical lines illustrate the optimal value of the LASSO tuning parameter (λ) for the intratumoral radiomic features. **(B)** A LASSO coefficient profile plot with different log(λ) values is displayed. The vertical dashed lines represent 9 intratumoral radiomic features with nonzero coefficients selected with the optimal λ value. **(C, D)** The same workflow was used for peritumoral radiomic feature analysis, as shown in **Figure 4A**, **(B, E, F)** The same workflow was used for the combined radiomics features, as shown in **Figures 4A, B**.

**Figure 6:**
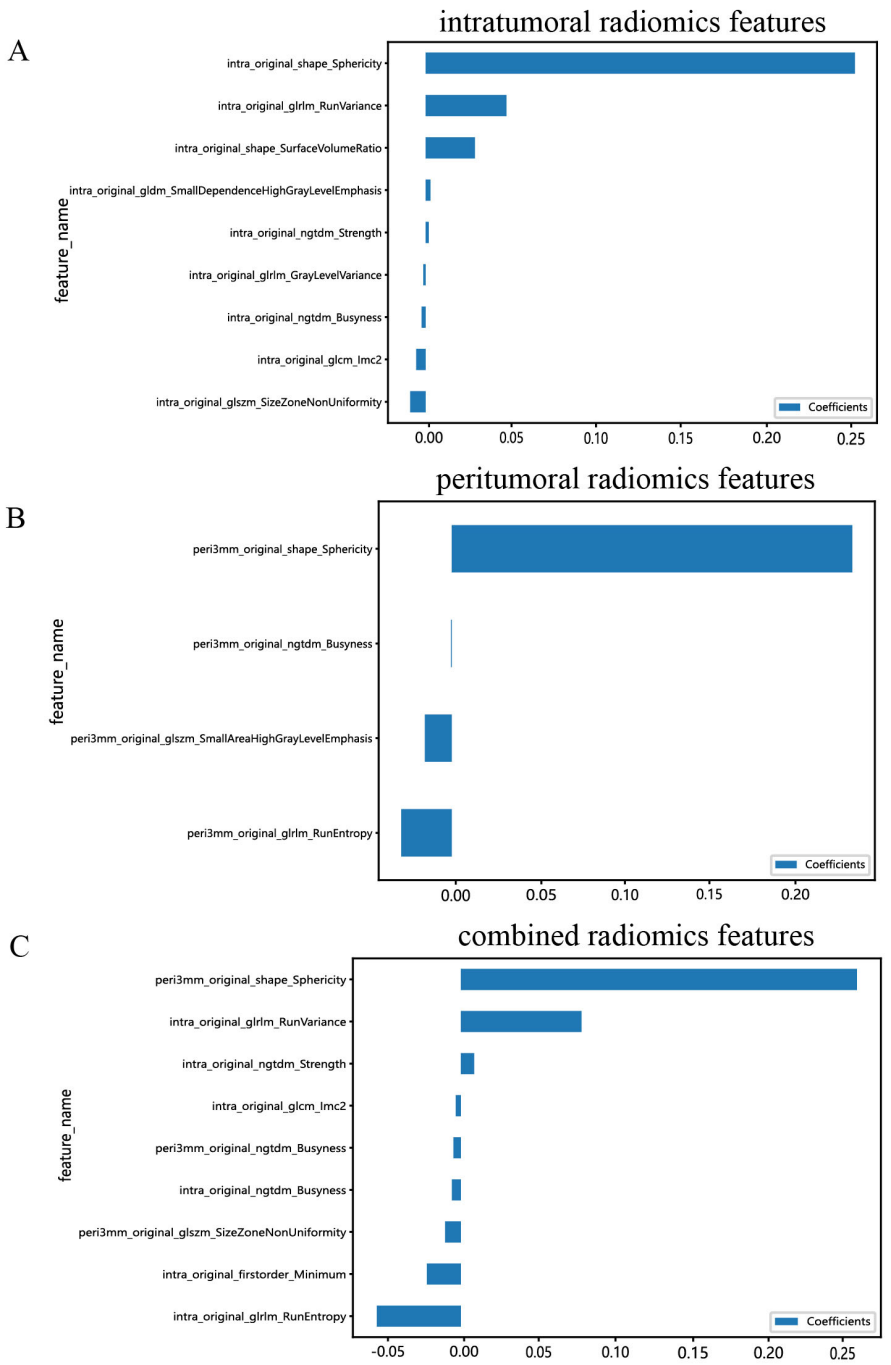
Bar graph of radiomic features that yielded nonzero values in the intratumoral **(A)**, peritumoral **(B)**, and combined regions **(C)**. (“intra” means “intratumoral”; “peri3 mm” means “peritumoral region with dilation of 3 mm”).

All the complete sets of intratumoral radiomic features (**Figure 4A**), peritumoral radiomic features (**Figure 4B**), and combined radiomic features (**Figure 4C**), along with their corresponding p values, are presented in **Figure 4**. Following feature selection using LASSO logistic regression, nine intratumoral radiomic features with nonzero coefficients were identified. The coefficients and mean standard errors (MSEs) obtained from a 10-fold validation were illustrated in **Figures 5A, B**. Similarly, four peritumoral radiomic features (**Figures 5C, D**), and nine combined radiomic features (**Figures 5E, F**) with nonzero coefficients were excluded. Of the nine retained combined radiomic features, four intratumoral features were identified using the intratumoral LASSO model, while two peritumoral features were obtained from the peritumoral LASSO model. Importantly, three of these retained features were novel: “peri3mm_original_glszm_SizeZoneNonUniformity,” “intra_original_firstorder_Minimum,” and “intra_original_glrlm_RunEntropy.” This finding suggests that the combined LASSO model may incorporate additional predictive information. The coefficients of these retained intratumoral, peritumoral, and combined radiomic features are displayed separately in **Figure 6**.

### Intratumoral radiomic models and performance

**Figure 7:**
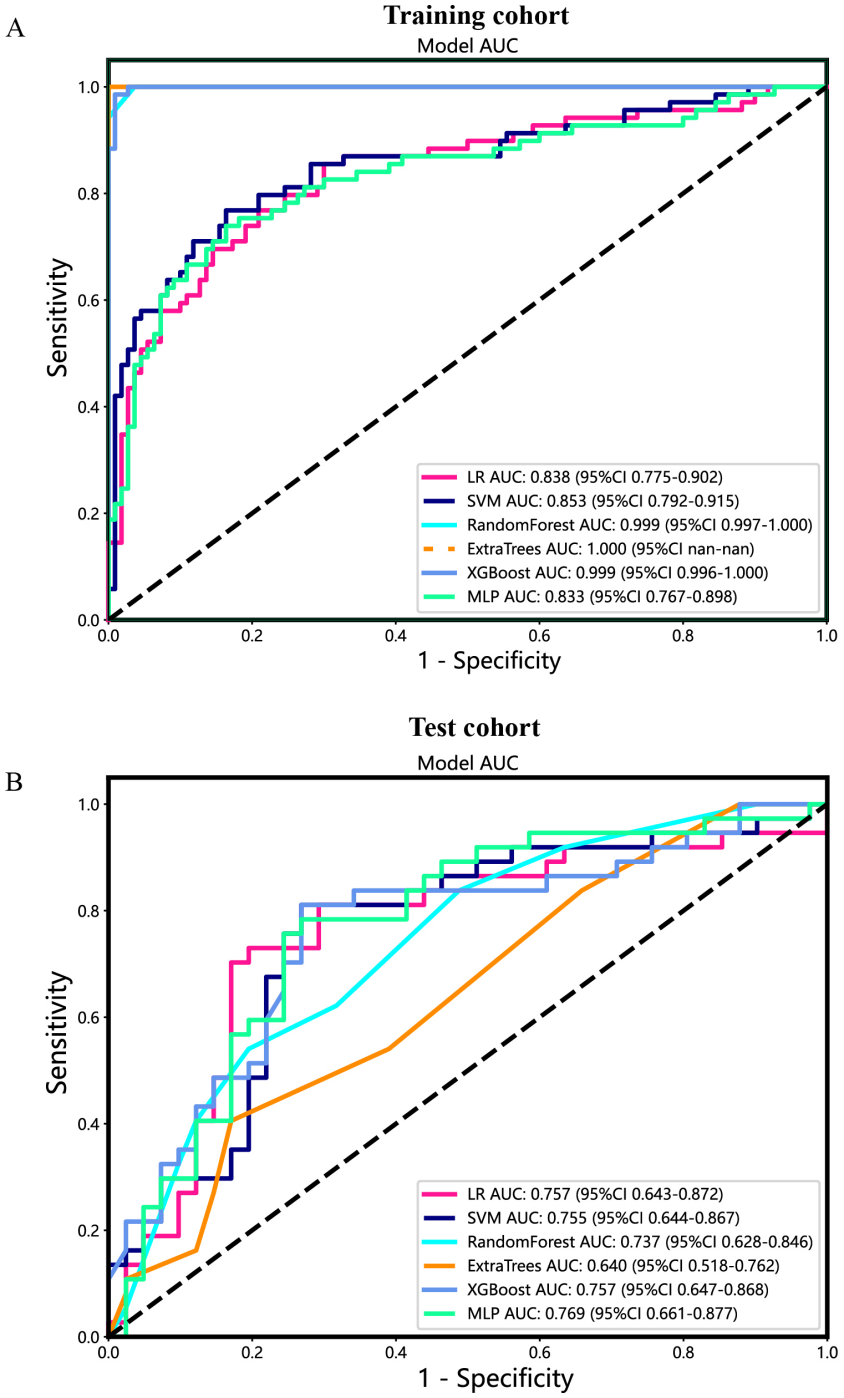
The ROC curves of different intratumoral radiomic models based on six machine learning algorithms for predicting PNETs. **(A)** The ROC curves of different intratumoral radiomic models in the training cohort. **(B)** The ROC curves of different intratumoral radiomic models in the test cohort.

Additionally, the ROC curves and AUCs of six intratumoral radiomics models generated using mainstream machine learning algorithms were presented in **Figure 7** for both the training and test cohorts. Moreover, additional details are displayed in **Table 2**. The RF, ExtraTrees, and XGBoost models tended to overfit the data. In contrast, the SVM model, with an AUC of 0.853 (95% CI 0.7920 - 0.9147) in the training cohort and an AUC of 0.755 (95% CI 0.6438 - 0.8671) in the test cohort, seemed to demonstrate the most appropriate performance and there was improved consistency between the training and test cohorts.

**Table 2:** Diagnostic performance of different models for predicting PNETs in training and test cohorts.

Model	Cohort	AUC (95% CI)	Accuracy	Sensitivity	Specificity	PPV	NPV
Intratumoral model (LR)	Training	0.838 (0.7752 - 0.9015)	0.777	0.754	0.791	0.693	0.837
	Test	0.757 (0.6432 - 0.8716)	0.756	0.703	0.805	0.765	0.750
Intratumoral model (RF)	Training	0.999 (0.9973 - 1.0000)	0.978	0.971	0.982	0.971	0.982
	Test	0.737 (0.6278 - 0.8462)	0.654	0.622	0.683	0.639	0.667
Intratumoral model (ExtraTrees)	Training	1.000 (1.0000 - 1.0000)	0.615	0.000	1.000	0.000	0.615
	Test	0.640 (0.5185 - 0.7617)	0.577	0.270	0.854	0.625	0.565
Intratumoral model (XGBoost)	Training	0.999 (0.9964 - 1.0000)	0.983	0.971	0.991	0.985	0.982
	Test	0.757 (0.6469 - 0.8679)	0.756	0.784	0.732	0.725	0.789
Intratumoral model (MLP)	Training	0.833 (0.7673 - 0.8984)	0.793	0.725	0.836	0.735	0.829
	Test	0.769 (0.6605 - 0.8767)	0.744	0.757	0.732	0.718	0.769
Intratumoral model (SVM*)	Training	0.853 (0.7920 - 0.9147)	0.804	0.754	0.836	0.743	0.844
	Test	0.755 (0.6438 - 0.8671)	0.756	0.784	0.732	0.725	0.789
Peritumoral model (SVM*)	Training	0.841 (0.7805 - 0.9024)	0.737	0.899	0.636	0.608	0.909
	Test	0.785 (0.6780 - 0.8922)	0.756	0.730	0.780	0.750	0.762
Combined model (SVM*)	Training	0.861 (0.7945 - 0.9162)	0.810	0.754	0.845	0.754	0.845
	Test	0.822 (0.7245 - 0.9066)	0.778	0.784	0.756	0.744	0.795

*Represents models were constructed based on SVM.

LR, logistic regression; SVM, support vector machine; RF, random forest; MLP, multilayer perceptron; XGBoost, extreme gradient boosting; CI, credibility interval.

Specifically, the SVM model demonstrated superior performance in the training cohort compared to the LR and MLP models. In the test cohort, the SVM model achieved an accuracy of 0.804, sensitivity of 0.754, specificity of 0.836, PPV of 0.743, and NPV of 0.844 (**Table 2**).

**Figure 8:**
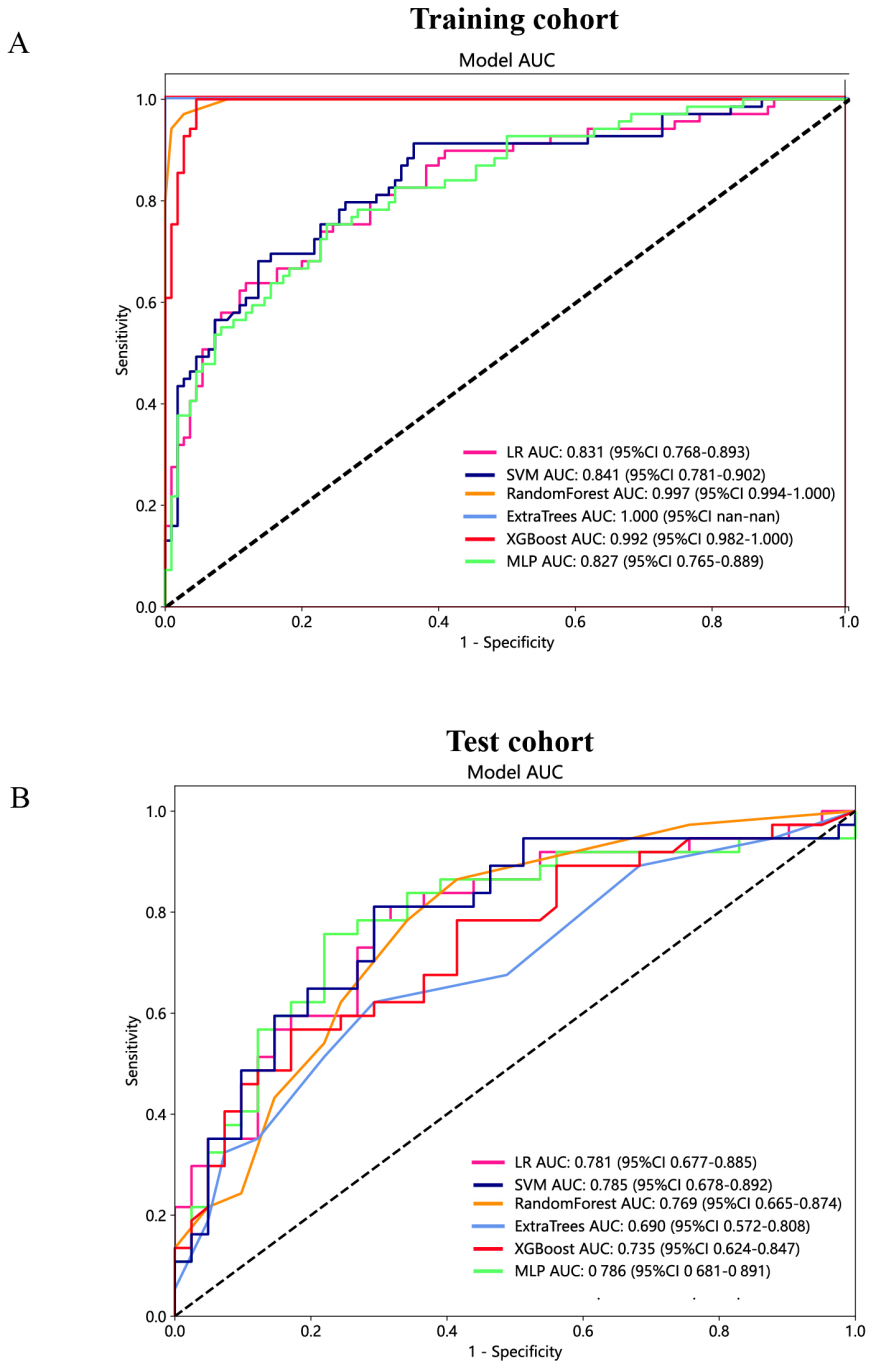
The ROC curves of different peritumoral radiomic models based on six machine learning algorithms for predicting PNETs. **(A)** The ROC curves of different peritumoral radiomic models in the training cohort. **(B)** The ROC curves of different peritumoral radiomic models in the test cohort.

Furthermore, based on the peritumoral radiomics, the SVM model also achieved an appropriate and consistent performance in both the training (**Figure 8A**) and test (**Figure 8B**) cohorts. Ultimately, to establish more stable and sustainable intratumoral, peritumoral, and combined radiomic models, the SVM model was deemed the most appropriate for further analysis and was chosen as the foundational algorithm.

### Construction and assessment of the peritumoral and combined radiomic models

**Figure 9:**
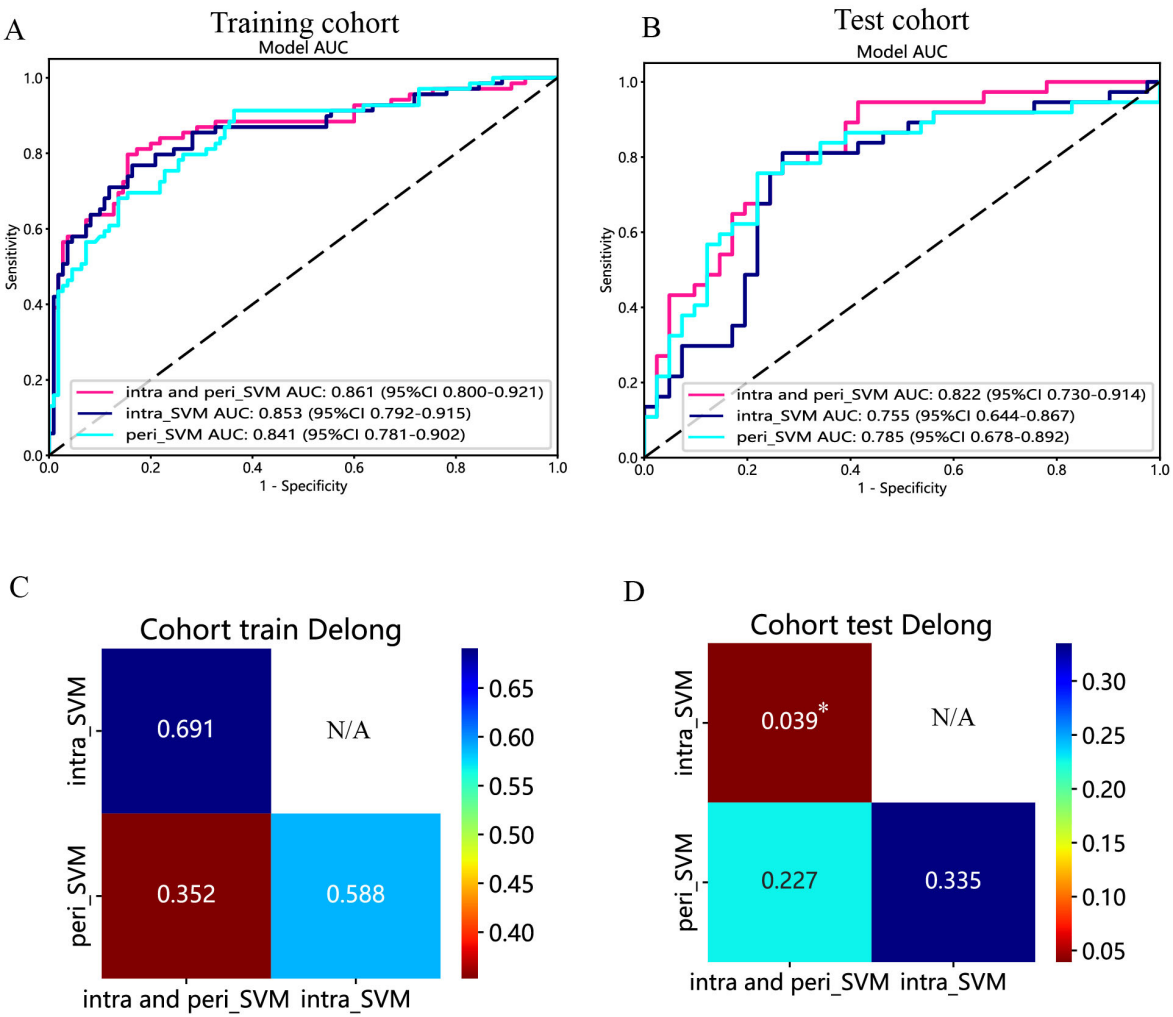
The ROC curves of the intratumoral radiomic model based on SVM (abbreviated “intra_SVM”), the peritumoral radiomic model based on SVM (abbreviated “peri_SVM”), and the combined radiomic model (abbreviated “intra- and peri_SVM”) in the **(A)** training cohort and **(B)** test cohort. The results of the Delong test in the **(C)** training and **(D)** test cohorts. (*indicates P < 0.05).

**Figure 10:**
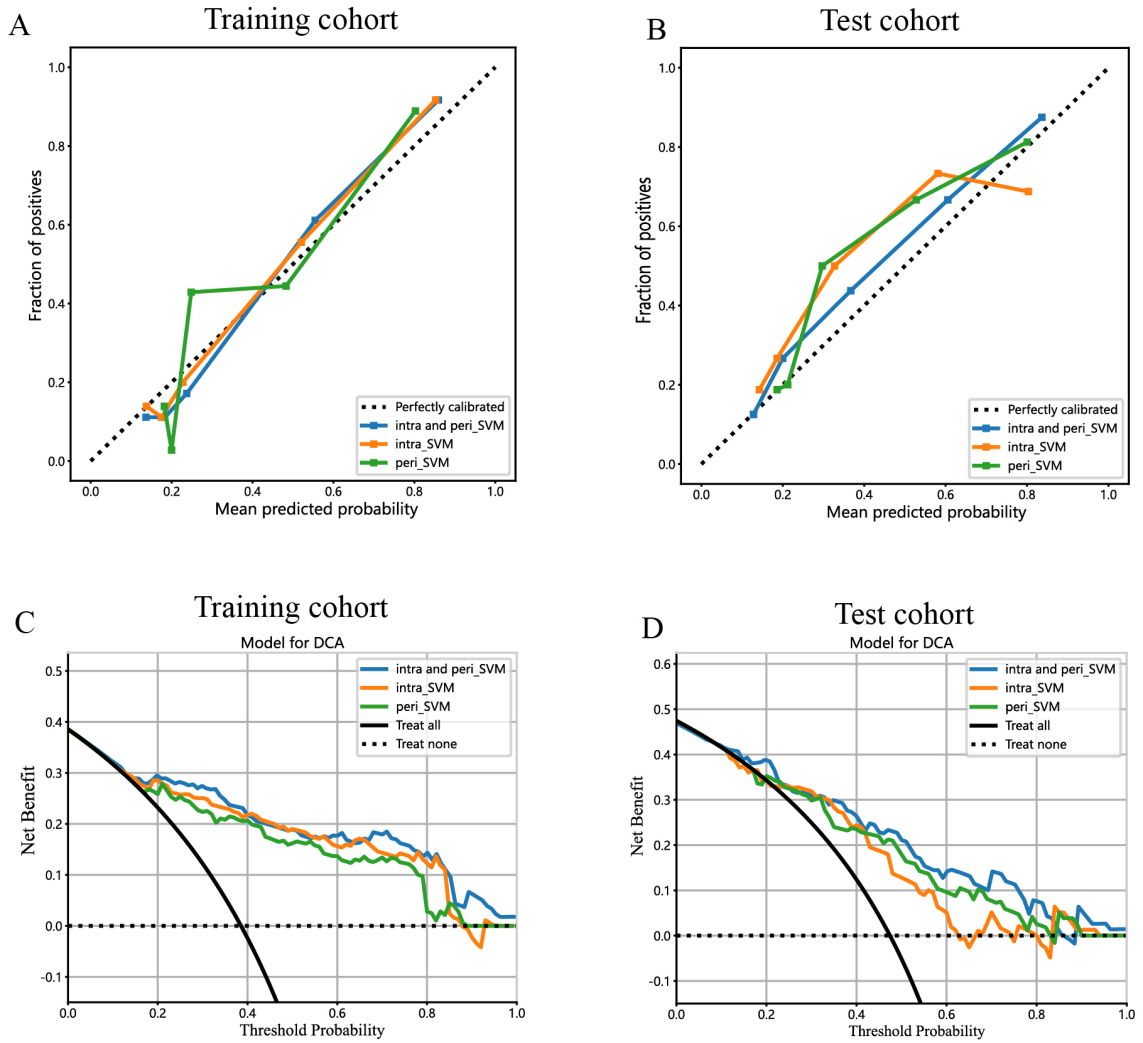
Calibration curves for the intratumoral radiomic model based on SVM (abbreviated “intra_SVM”), peritumoral radiomic model based on SVM (abbreviated “peri_SVM”), and combined radiomic model (abbreviated “intra and peri_SVM”) in the **(A)** training cohort and **(B)** test cohort. The DCA curves for the intratumoral, peritumoral, and combined radiomics models based on SVM in the training **(C, D)** test cohorts.

The predictive performance of the peritumoral and combined radiomic SVM models in both the training and test cohorts is summarized in **Table 2**. The ROC curves for the intratumoral radiomic model, peritumoral radiomic model, and combined radiomic model are illustrated in **Figure 9** for both the training (**Figure 9A**) and test cohorts (**Figure 9B**). Among these three radiomic models, the combined radiomic model achieved the optimal performance, with the highest AUC in both the training (AUC=0.861) and test (AUC=0.822) cohorts. Furthermore, the combined radiomic model achieved a superior accuracy of 0.810 in the training cohort and 0.778 in the test cohort (**Table 2**). To objectively assess the effectiveness of these models, the Delong test was conducted. In the training cohort, there was no statistically significant difference in the AUC between these three models (**Figure 9C**). Additionally, the AUC of the peritumoral radiomic model was consistent with that of the intratumoral radiomic model (peritumoral vs. intratumoral: AUC=0.785 vs. 0.755, p=0.335) (**Table 2**, **Figure 9D**) in the test cohort, demonstrating that the efficacy of the peritumoral model was not inferior to that of the intratumoral model. In contrast, the AUC of the combined model demonstrated statistically significant superiority over the other models in the test cohort, with AUC values of 0.822 compared to 0.755 for intratumoral models (p=0.039) and 0.822 compared to 0.785 for peritumoral models (p=0.227) (**Table 2**, **Figure 9D**). This suggests that the combined model might exhibit the highest diagnostic efficacy. Furthermore, the calibration curves of the combined model showed consistency between predicted and observed PNETs in both the training and test cohorts. The results of the H-L test indicated that the combined model had superior prediction accuracy (**Table 3**). The calibration curves for the training and test cohorts are presented in **Figures 10A, B**.

**Table 3:** The results of Hosmer-Lemeshow test.

Model	*P*-value	
	Training cohort	Test cohort
Intratumoral model	0.578	0.118
Peritumoral model	0.089	0.226
Combined model	0.175	0.759

Finally, DCA was performed to evaluate the performance of each model, with the results shown in **Figures 10C, D**.

**Figure 11:**
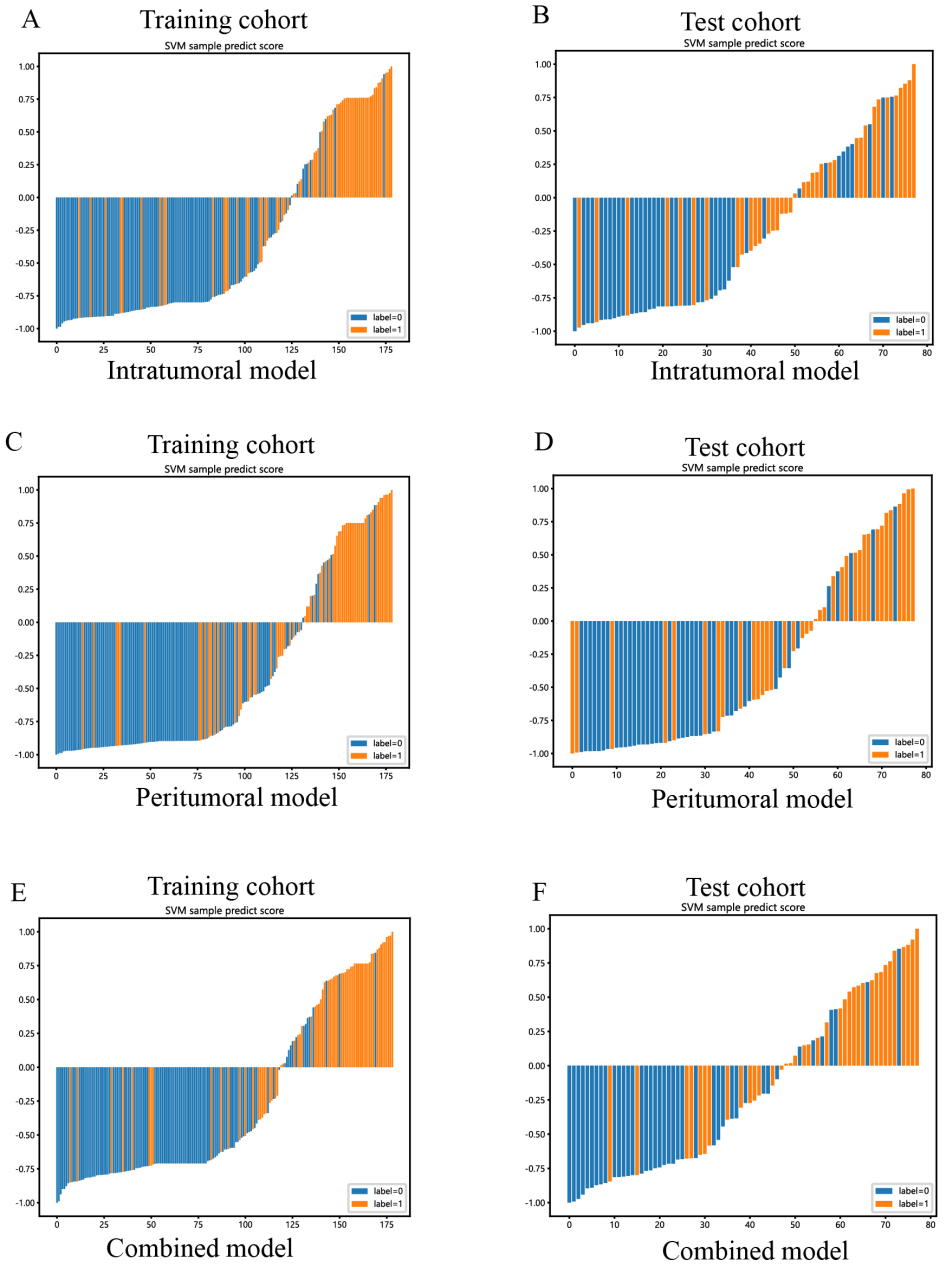
SVM-based prediction scores of the intratumoral **(A, B)**, peritumoral **(C, D)**, and combined **(E, F)** radiomics models in the training and test cohorts. (“label=0” means “pancreatic cancer”; “label=1” means “PNETs”).

The combined model showed a significantly higher net benefit for intervening with patients based on its prediction probability compared to hypothetical scenarios where no prediction model was available, such as treat-all or treat-none strategies. Additionally, the combined model demonstrated higher values in both the training and test cohorts compared to the other models. Therefore, the integration of this combined model appears to have the potential to improve the clinical effectiveness of preoperatively predicting PNETs before surgery and EUS-FNA/B procedures. The prediction scores of the intratumoral, peritumoral, and combined models are shown in **Figure 11**. Finally, this combined model was defined as radiomics signature.

### Construction of clinical signature and nomogram

**Figure 12:**
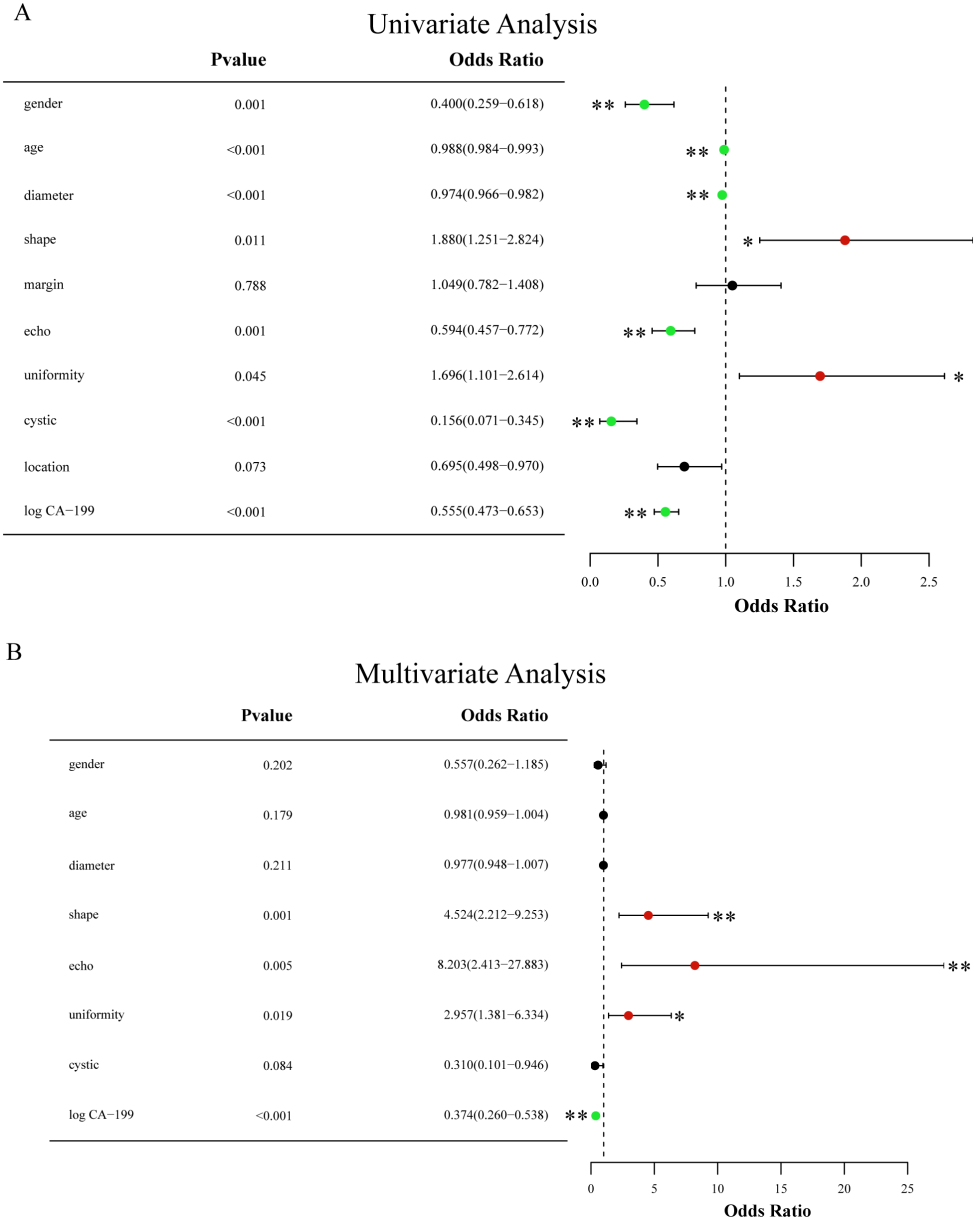
**(A)** Forest map of univariate logistic regression of clinical and EUS characteristics; **(B)** Forest map of multivariate logistic regression of clinical and EUS characteristics. * means P<0.05; ** means P<0.01.

**Figure 13:**
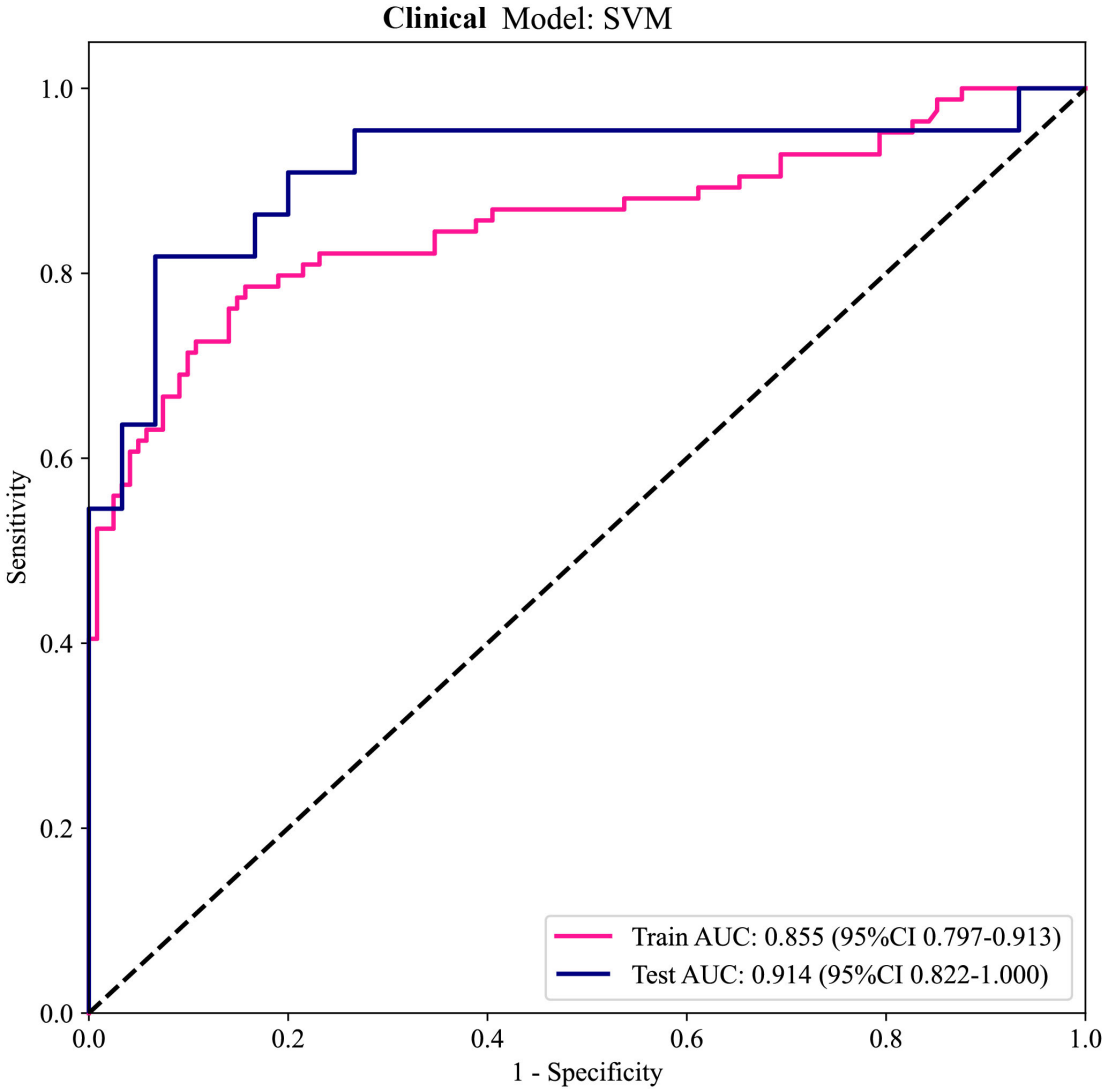
The ROC curves of the clinical signature in both the training and test cohorts.

**Figure 14:**
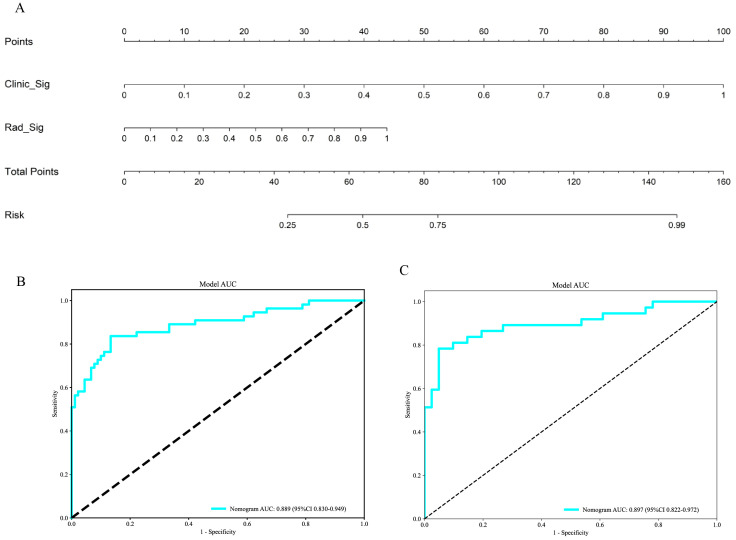
**(A)** The Nomogram for predicting PNETs based on radiomics signature (Rad_Sig) and clinical signature (Clinic_Sig). **(B)** The ROC curve of the nomogram in the training cohort. **(C)** The ROC curve of the nomogram in the test cohort.

**Figure 15:**
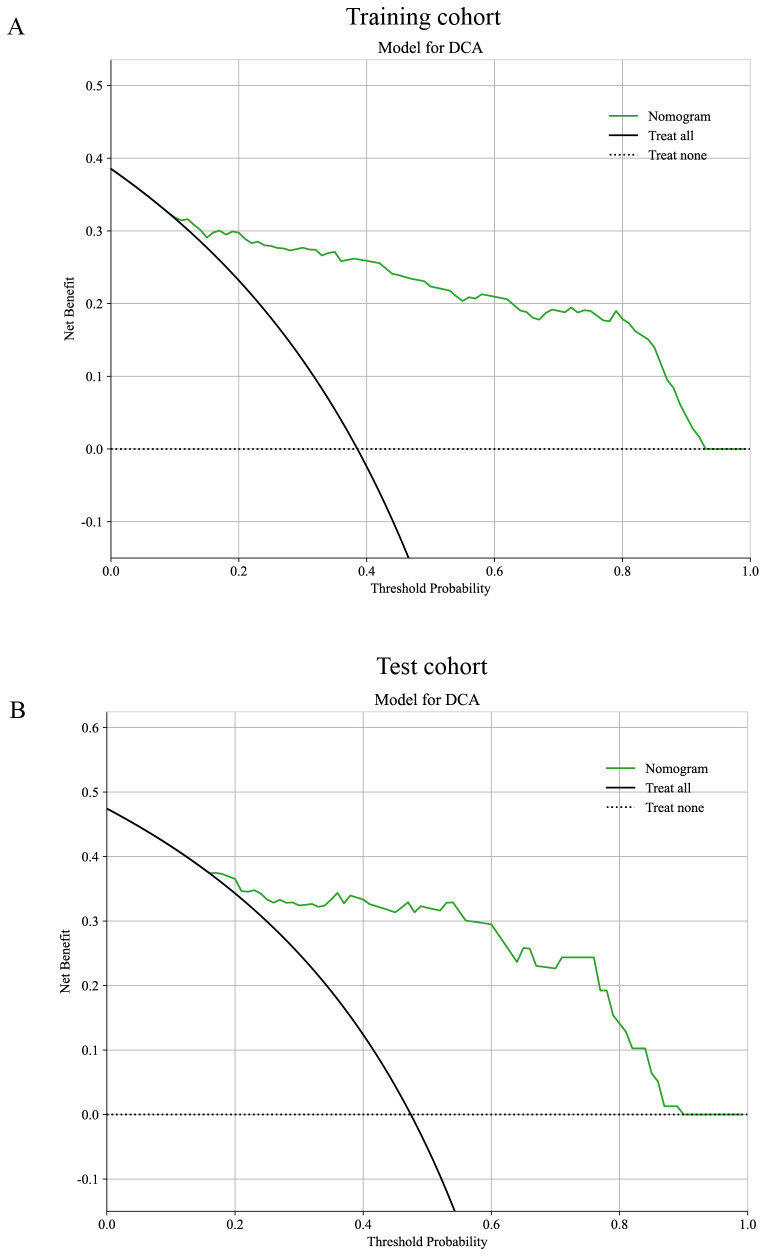
The DCA curves for the nomogram in the training **(A, B)** test cohorts.

The outcomes of the univariate (**Figure 12A**) and multivariable (**Figure 12B**) logistic regression analyses demonstrated that shape, echo characteristics, and CA-199 levels independently predicted the presence of PNETs. Subsequently, these parameters were utilized to develop a clinical signature using the SVM algorithm. The clinical signature attained a ROC value of 0.855 in the training cohort and 0.914 in the test cohort (**Figure 13**). A nomogram model (**Figure 14A**) was utilized to synthesize radiomic and clinical signatures, thereby enhancing predictive accuracy and consistency. This approach yielded an optimal performance, as evidenced by a ROC value of 0.897 in both the training (**Figure 14B**) and test (**Figure 14C**) cohorts. The nomogram demonstrated significantly superior performance compared to the clinical signature within the training cohort (Delong test, P=0.015), and exhibited consistency with the radiomics signature in both the training (Delong test, P=0.161) and test cohorts (Delong test, P=0.066). The DCA indicated that the nomogram demonstrated a substantial net clinical benefit in both the training cohort (**Figure 15A**) and the test cohort (**Figure 15B**). The calibration curves of the nomogram and clinical signature are detailed in **Supplementary Figure 4**.

## Discussion

In this research, we applied EUS images and assessed intratumoral and peritumoral regions to distinguish PNETs from pancreatic cancer. The combined radiomic model derived from integrating the features of the intratumoral and peritumoral regions achieved optimal prediction performance. These findings indicate that peritumoral regions may possess specific characteristics and contribute to the diagnosis of PNETs. Previous studies have revealed the effectiveness of EUS imaging-based radiomic, machine learning, and deep learning approaches in predicting gastrointestinal stromal tumors and pancreatic ductal adenocarcinoma (14, 25, 26). Similarly, Huang J reported that the combined nomogram model, based on deep learning contrast-enhanced ultrasound and clinical features, performed great preoperatively differentiating the aggressiveness of PNETs (17). Our prior research demonstrated that integrating an EUS radiomics signature with a clinical signature enhanced the predictive performance of models distinguishing PNETs from pancreatic cancer (27). However, there is a lack of studies evaluating the utility of EUS imaging-based intratumoral and/or peritumoral radiomics for predicting PNETs in this context.

The ability of EUS to conduct comprehensive ultrasound scans of the entire pancreas from the intragastric and intraduodenal positions, with proximity and minimal interference, has been widely acknowledged (28). This capability enables the generation of high-resolution EUS images, facilitates the visualization of intricate anatomical features, and promotes EUS as the superior approach for detecting small pancreatic lesions compared with traditional CT and MRI (10, 29). Additionally, the ability to obtain tissue samples through EUS-FNA/B enhances the diagnostic precision of EUS, establishing it as a dependable method for diagnosing PNETs (30, 31).

Preoperative EUS imaging for functional PNETs can accurately determine the relationship and proximity of the lesion to the MPD, thereby influencing the therapeutic decisions in choosing the proper surgical treatment, whether radical or local resection (32). Notably, a multicenter study revealed the superior diagnostic capabilities of non-contrast MRI radiomic models and combined models for predicting Grade 1 and 2/3 nonfunctional PNETs, surpassing the performance of clinical and radiological feature models (18). Similarly, Gu D demonstrated that radiomic signatures derived from arterial and portal venous phase CT images were more likely to accurately predict the histologic grade of PNETs (33). A previous study suggested volumetric CT texture characteristics as a quantitative tool to differentiate atypical PNETs from pancreatic ductal adenocarcinoma (34).

Multiple mainstream machine learning algorithms were employed concurrently to develop an optimal two-class prediction EUS imaging-based radiomic model for distinguishing PNETs from pancreatic cancer, addressing the shortcomings of individual algorithms. The SVM algorithm exhibited superior accuracy and consistency in this context, leading to its selection for further model refinement. The SVM algorithm exhibited superior accuracy and consistency in this context, leading to its selection for further model refinement.

The results demonstrated that the intratumoral radiomic model was effective in differentiating PNETs from pancreatic cancer, with an AUC of 0.853 (95% CI 0.7920-0.9147) in the training cohort and an AUC of 0.755 (95% CI 0.6438-0.8671) in the test cohort. The subjective assessment of EUS image characteristics, such as tumor size, shape, border, and vascular invasion, is heavily dependent on the operator’s expertise, leading to insufficient consistency (35, 36). Consequently, our findings, derived from high-throughput EUS radiomics features and machine learning, may provide potential novel and objective insight into improving the ability of EUS to predict PNETs.

The existing radiomic literature about PNETs focuses on the intratumoral region, not the peritumoral region (16, 18, 37). Correspondingly, many previous studies have demonstrated the good performance of the peritumoral radiomic model for various tumors in terms of pathological outcomes, lymph node metastasis, and recurrence risk stratification. Xie N reported that the evaluation accuracy of the peritumoral multiparametric MRI radiomic feature model for predicting pancreatic cancer pathological outcomes was marginally greater than that of intratumoral features in the training cohort (22). The integration of intratumoral and peritumoral features could enhance the efficiency of predicting tumor recurrence in intrahepatic cholangiocarcinoma patients (38). Shi JX proposed that the integration of intratumoral and peritumoral radiomic features extracted from MRI images demonstrated notable efficacy in the prediction of lymph node metastasis in early-stage cervical cancer (39). In terms of biological and clinical characteristics, PNETs differ significantly from pancreatic cancer, such as tissue origin, symptoms, risk of lymph node metastasis, prognosis, treatment strategies, and molecular biological characteristics (40–42).

Our prior research demonstrated that integrating EUS intratumoral features with radiomic features of the peritumoral region, extending 3 mm outward enhanced the model’s efficacy in distinguishing between insulinomas and nonfunctional PNETs (43). However, it remains unclear whether the peritumoral region of PNETs contains predictive and diagnostic information. In our study, we constructed and validated a peritumoral radiomic model for predicting PNETs. In comparison to the peritumoral feature models with expansions of 1mm and 5mm, the model incorporating a 3mm expansion exhibited an AUC of 0.841 in the training cohort and an AUC of 0.785 in the test cohort. Importantly, the comparison of AUC values between the intratumoral and peritumoral models did not yield statistically significant differences (Delong p > 0.05).

From our perspective, both the peritumoral and intratumoral regions possess similar potential for distinguishing PNETs from pancreatic cancer and may exhibit synergistic effects in this task. Therefore, to fully utilize the data extracted from different regions, a combined model derived from both peritumoral and intratumoral radiomic features was established and assessed. In an intriguing development, while the efficacy of the combined radiomics model was comparable to that of the intratumoral and peritumoral models within the training cohort, it demonstrated significantly superior performance in the test cohort, achieving an AUC of 0.822 (95% CI: 0.7245 - 0.9066) compared to the intratumoral model. Moreover, this combined radiomics model appeared to exhibit the highest levels of accuracy, specificity, NPV, and PPV in both the training and test cohorts, as detailed in **Table 3**. These findings underscore the substantial capability of the combined model to predict tumor efficacy, attributed to the enhancement effect of peritumoral radiomic features on the intratumoral model. Furthermore, the DeLong test and H-L test provided validation of the superior accuracy and validity of the combined model. These results align with findings from various prior studies examining different types of tumors (44–46). Sun Q’s research indicated that the integration of intratumoral and peritumoral areas led to notably improved predictive capabilities for both radiomic and deep learning models in the context of forecasting axillary lymph node metastasis in individuals with breast cancer (47). This evidence suggests that the peritumoral region, particularly the tumor-adjacent parenchyma surrounding tumor lesions, may offer predictive information for PNETs. Finally, we constructed a nomogram, integrating radiomics and clinical signatures, which also achieved an excellent performance.

Despite the notable efficacy of EUS imaging demonstrated by the combined intratumoral and peritumoral models, it is important to note that this study has limitations. Notably, it was a retrospective analysis conducted at a single institution, potentially introducing selection bias. Additionally, in image segmentation, all definitions of boundaries are derived from manual segmentation, making bias inevitable (48).

Furthermore, it is important to note that the study exclusively employed conventional EUS imaging, thereby neglecting the potential advantages offered by contrast-enhanced EUS and elastography EUS techniques (49–51). The biological mechanisms underlying the features of the peritumoral region remain inadequately understood. Additionally, incorporating a comparative analysis of the healthy parenchyma situated at a greater distance from the tumor in the two patient cohorts as a control test could provide valuable insights.

Although the machine learning model, integrating intratumoral and peritumoral radiomic features from EUS imaging, demonstrated significant efficacy, this study is constrained by several limitations. Retrospective analyses conducted at a single center, with a small sample size, are susceptible to selection bias, and the manual segmentation process may introduce additional bias in image segmentation. The study spanned a long period (October 2012 to October 2023), during which improvements in imaging technology might have influenced the quality and consistency of EUS images. Furthermore, this study exclusively concentrated on the intratumoral and peritumoral radiomic features of pancreatic lesions, omitting clinical characteristics such as tumor size, location, tumor markers, and blood glucose levels. Therefore, conducting further research on EUS-based radiomics for PNETs that involve multiple centers, large sample sizes, prospective designs, and multimodal approaches is crucial. Furthermore, the utilization of deep learning techniques and exploration of underlying biological changes in peritumor imaging features could be utilized to reduce bias and improve the interpretability of the models.

## Conclusion

In conclusion, a proficient EUS-based radiomic model integrating intratumoral and peritumoral radiomic features was suggested and confirmed to effectively differentiate PNETs from pancreatic cancer. Among the various machine learning algorithms, the combined model applying SVM achieved the optimal diagnostic performance and might provide potential novel insight into improving the clinical application of EUS in predicting PNETs.

## Data Availability

The original contributions presented in the study are included in the article/**Supplementary Material**. Further inquiries can be directed to the corresponding authors.
