# Supplementary material for: EUS-based intratumoral and peritumoral machine learning radiomics analysis for distinguishing pancreatic neuroendocrine tumors from pancreatic cancer
Source: Front Oncol. 2025 Mar 4;15:1442209. doi: 10.3389/fonc.2025.1442209 (PMC11913666; doi:10.3389/fonc.2025.1442209)
Supplement: Supplementary file 2 [file DataSheet2.pdf]

hyperparameter

```
models['LR'] = LogisticRegression(random_state=0)
models['NaiveBayes'] = GaussianNB()
models['SVM'] = SVC(probability=True, random_state=0)
models['KNN'] = KNeighborsClassifier(algorithm='kd_tree')
models['DecisionTree'] = DecisionTreeClassifier(max_depth=None,
min_samples_split=2, random_state=0)
models['RandomForest'] = RandomForestClassifier(n_estimators=10,
max_depth=None,
                                                min_samples_split=2,
random_state=0)
models['ExtraTrees'] = ExtraTreesClassifier(n_estimators=10, max_depth=None,
                                            min_samples_split=2,
random_state=0)
models['XGBoost'] = XGBClassifier(n_estimators=10,
objective='binary:logistic',
                                  use_label_encoder=False,
eval_metric='error')
models['LightGBM'] = LGBMClassifier(n_estimators=10, max_depth=-1,
objective='binary')
models['GradientBoosting'] = GradientBoostingClassifier(n_estimators=10,
random_state=0)
models['AdaBoost'] = AdaBoostClassifier(n_estimators=10, random_state=0)
models['MLP'] = MLPClassifier(hidden_layer_sizes=(128, 64, 32), max_iter=300,
solver='sgd',
                              random_state=0)
```

None overfit

```
models['LR'] = LogisticRegression(random_state=0)
models['NaiveBayes'] = GaussianNB()
models['SVM'] = SVC(probability=True, random_state=0)
models['KNN'] = KNeighborsClassifier(algorithm='kd_tree')
models['DecisionTree'] = DecisionTreeClassifier(max_depth=3,
min_samples_split=2, random_state=0)
models['RandomForest'] = RandomForestClassifier(n_estimators=10, max_depth=3,
                                                min_samples_split=2,
random_state=0)
models['ExtraTrees'] = ExtraTreesClassifier(n_estimators=10, max_depth=3,
```

```
                                            min_samples_split=2,
random_state=0)
models['XGBoost'] = XGBClassifier(n_estimators=10,
objective='binary:logistic', max_depth=3,
                                  use_label_encoder=False,
eval_metric='error')
models['LightGBM'] = LGBMClassifier(n_estimators=10, max_depth=3,
objective='binary')
models['GradientBoosting'] = GradientBoostingClassifier(n_estimators=10,
random_state=0, max_depth=3)
models['AdaBoost'] = AdaBoostClassifier(n_estimators=10, random_state=0)
models['MLP'] = MLPClassifier(hidden_layer_sizes=(128, 64, 32), max_iter=300,
solver='sgd',
                              random_state=0)
```
