# Supplementary material for: EUS-based intratumoral and peritumoral machine learning radiomics analysis for distinguishing pancreatic neuroendocrine tumors from pancreatic cancer
Source: Front Oncol. 2025 Mar 4;15:1442209. doi: 10.3389/fonc.2025.1442209 (PMC11913666; doi:10.3389/fonc.2025.1442209)
Supplement: Supplementary file 4 [file DataSheet4.pdf]

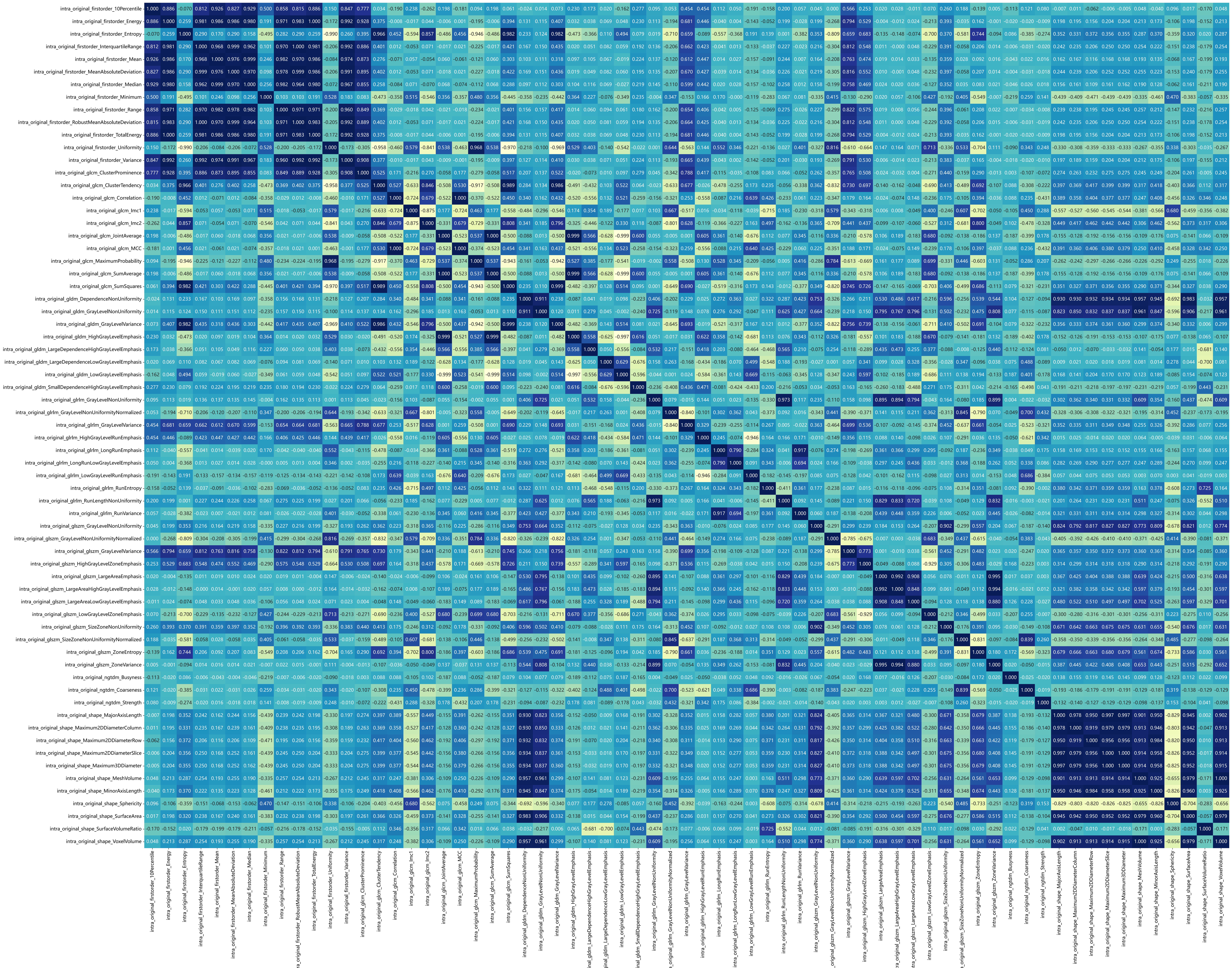

Spearman's correlation coefficient of Intratumoral radiomics features
