# Supplementary figures and images for: EUS-based intratumoral and peritumoral machine learning radiomics analysis for distinguishing pancreatic neuroendocrine tumors from pancreatic cancer
Source: Front Oncol. 2025 Mar 4;15:1442209. doi: 10.3389/fonc.2025.1442209 (PMC11913666; doi:10.3389/fonc.2025.1442209)

A

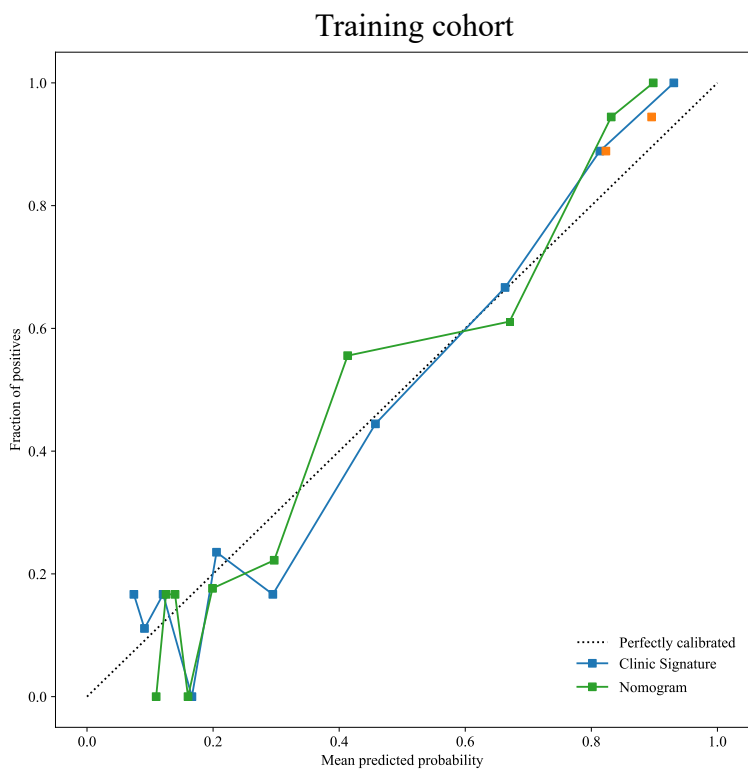

B

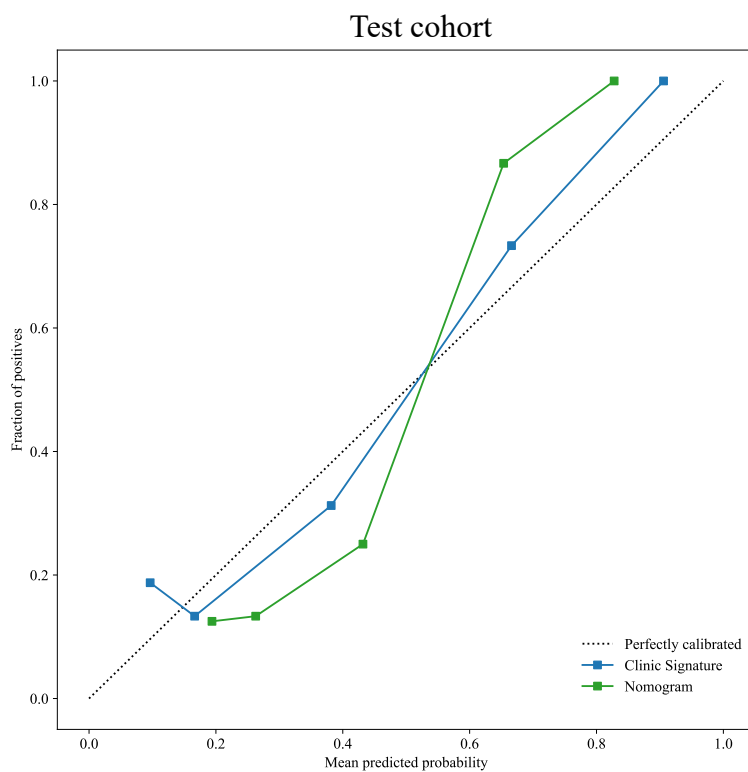

Supplement: Supplementary file 7 [file DataSheet7.pdf]
